# Resorcin

**Published:** 1890-07-05

**Authors:** 


### THERAPEUTICS.

### RESORCIN.

We have already alluded to the promise of a permanent reputation held out by this drug, which combines with undoubted activity, advantages possessed by none of its congeners. It is practically non-poisonous, and being rapidly eliminated from the system may be taken continuously for long periods without the risk of cumulative effects. It is soluble in all ordinary vehicles, and is so volatile that its vapour mixed with air may be inhaled without inconvenience, though a powerful antiseptic and germicide. Coagulating fibrin and albumen, it is also hæmostatic. Drs. Le Blond and Baudier have for the last ten years used a 10 per cent. solution of resorcin in glycerine in the treatment of ordinary cases of diphtheria, painting the fauces, &c., every hour or two hours. It seemed to be rapidly absorbed, for the enlargement of the glands was generally diminished within 24 hours and had entirely disappeared after two days of such treatment. At the same time they saturated the air of the room by means of a Lister's spray apparatus with a watery solution of 50 grams to the litre, *i.e.*, 5 per cent. In nasal cases the nares were first rendered insensitive to the irritation of the glycerine by the injection of a 1 per cent. sol. of cocaine, but for laryngeal diphtheria, or so-called croup, this treatment was not available, since the parts affected could not be reached with the brush.

